# T cell phenotype and clonality changes in myeloma patients with short overall survival

**DOI:** 10.1172/jci.insight.181096

**Published:** 2025-04-22

**Authors:** Alenka Djarmila Behsen, Esten Nymoen Vandsemb, Tobias Schmidt Slørdahl, Karen Dybkær, Maja Zimmer Jakobsen, Muhammad Kashif, Johan Lund, Vincent Luong, Astrid Marta Olsnes, Anders Waage, Anne Marit Sponaas, Kristine Misund

**Affiliations:** 1 Department of Clinical and Molecular Medicine, Norwegian University of Science and Technology, Trondheim, Norway.; 2 Department of Hematology, St. Olavs Hospital, Trondheim, Norway.; 3 Department of Hematology, Aalborg University Hospital, Aalborg, Denmark.; 4 Department of Clinical Medicine, Aalborg University, Aalborg, Denmark.; 5 Department of Medicine, Center for Hematology and Regenerative Medicine, Karolinska Institutet, Stockholm, Sweden.; 6 Department of Hematology, Karolinska University Hospital, Stockholm, Sweden.; 7 Section for Hematology, Department of Medicine, Haukeland University Hospital, Bergen, Norway.; 8 Institute of Clinical Science, University of Bergen, Norway.; 9 Biobank1, St. Olavs Hospital, Trondheim, Norway.; 10 Department of Medical Genetics, St. Olavs Hospital, Trondheim, Norway.

**Keywords:** Hematology, Immunology, Oncology, Cancer, T cell receptor, T cells

## Abstract

Overall survival (OS) in multiple myeloma (MM) varies between a couple of months to more than 20 years, influenced by tumor characteristics, the tumor microenvironment (TME), and patient factors such as age and frailty. We analyzed sequential BM samples from 45 MM patients with OS less than 3 years versus more than 8 years using mass cytometry and bulk TCRβ sequencing. Patients with long OS demonstrated stability in the TME and T cell environments, while those with short OS had significant changes at relapse, including fewer T cells, increased Tregs, and more activated and exhausted CD8^+^ T cells. Notably, higher programmed cell death 1 expression in CD8^+^ T cells at diagnosis correlated with short OS. Additionally, short-OS patients exhibited a more monoclonal T cell environment at relapse, with abundance of hyperexpanded clones. These findings reveal distinct immune cell differences between patients with short and long OS.

## Abstract 2

Shorter survival in myeloma patients linked to T-cell changes, including fewer T cells and hyperexpanded T cell clones.

## Introduction

Multiple myeloma (MM) is a hematological malignancy of the plasma cells (PCs) causing their accumulation in the BM. This is accompanied by clinical manifestations such as anemia, bone destruction, hypercalcemia, kidney failure, and frequent infections, the latter being a leading cause of morbidity and death in patients with MM (1).

Our understanding of the immune system and its impact on MM has greatly expanded over the last 2 decades. Patients with myeloma have a dysregulated and suppressed immune system, most apparent as immunoparesis, which has been well known for many years and directly affects the tendency of infections (2). In addition to the reduction of normal PCs, changes in innate and other adaptive immune cells have been demonstrated. Studies have shown a switch to immunosuppressive macrophages and DCs, increase in Tregs (3) and myeloid-derived suppressor cells (MDSCs) (4, 5), impaired NK cells (6), and loss of T cell function in the tumor microenvironment (TME) because of exhaustion and senescence (7). Studies have also compared the TME in monoclonal gammopathy of undetermined significance (MGUS), smoldering MM, and MM. For instance, loss of stem-like memory T cells in the transition from MGUS to MM has been demonstrated (8).

**Figure 1:**
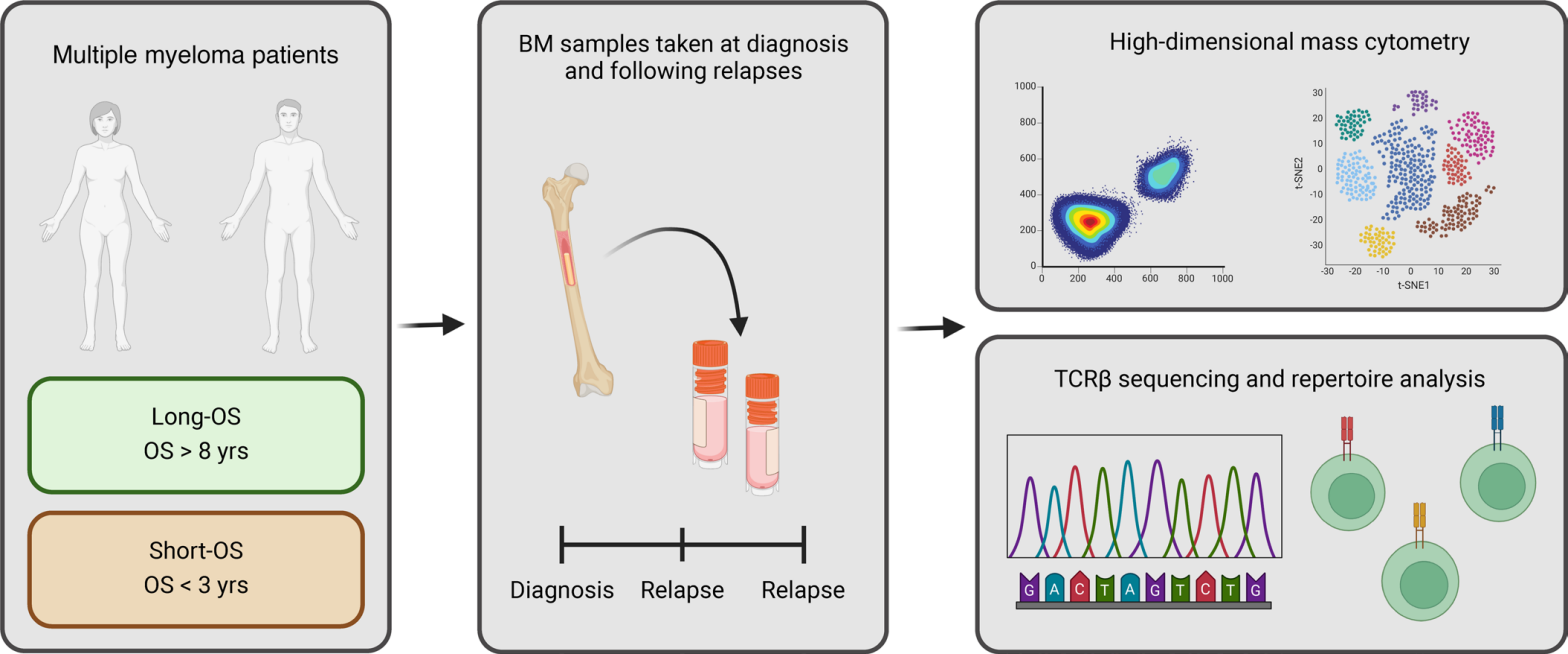
Overview of research approach. BM samples, from either only diagnosis or paired from diagnosis and relapse, were obtained from long-OS (OS > 8 years) and short-OS (OS < 3 years) myeloma patients. Samples were analyzed using high-dimensional mass cytometry and bulk TCRβ sequencing. Created with BioRender.com.

Median survival in unselected groups of patients with MM is 5 to 7 years. However, there is large variation from a couple of months to more than 20 years, which probably is related to variations in intrinsic tumor characteristics as well as their interactions with the TME (9). Comparing groups of patients with very different survival might provide new insight into factors determining disease outcome. Such considerations motivated the design of our TME study where we compared sequential BM samples from a cohort of 45 MM patients with overall survival (OS) less than 3 years versus more than 8 years (Table 1). We used high-dimensional mass cytometry analysis and bulk T cell receptor β sequencing (TCR-Seq) to capture the diversity and functionality in the TME and the variety of T cell populations (Figure 1). Here, we demonstrated that the TME in long-OS patients is relatively stable from diagnosis to relapse, while there are significant changes in the T cell compartment of short-OS patients at relapse. We observed a decrease in T cells in short-OS patients and increased expression of markers for activation and exhaustion and abundance of hyperexpanded T cell clones.

## Results

**Figure 2:**
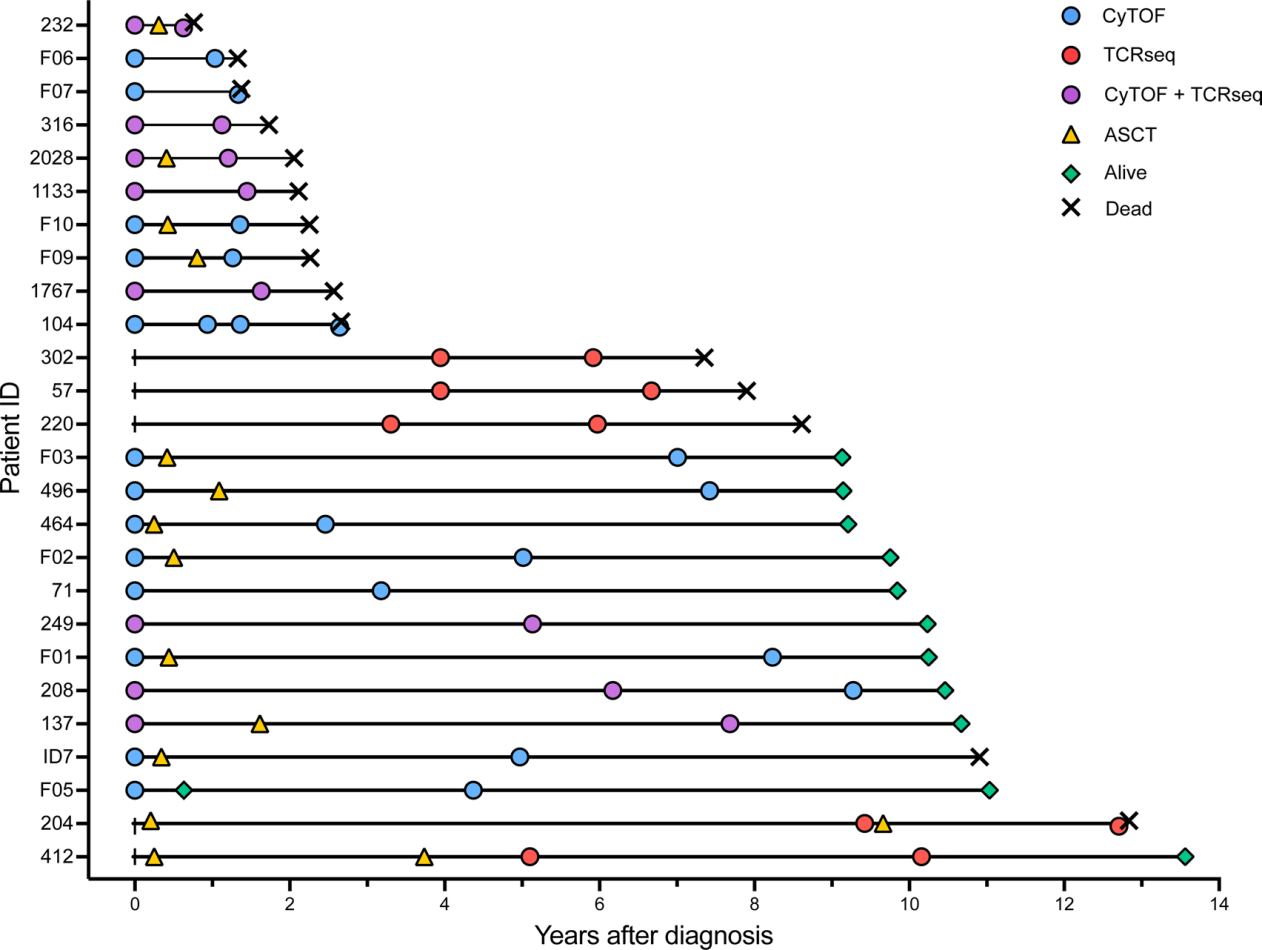
Swimmer plot showing time points for sample collection and immune assay performed in relation to OS. Includes only patients with paired samples. Patients with only samples from diagnosis are shown in Supplemental Figure 5. CyTOF was performed on CD138-negative bone marrow mononuclear cells (BMMNCs) or BMMNCs; bulk TCRβ sequencing (TCR-Seq) was performed on CD138-negative BMMNCs. Autologous stem cell transplant (ASCT) is also included and denotes time point for reinfusion of stem cells.

To investigate the immune composition, its development from diagnosis to relapse in patients with MM, and how this may differ in patients with short and long survival, we performed mass cytometry analysis using a custom panel of 37 targets (Supplemental Table 1; supplemental material available online with this article; https://doi.org/10.1172/jci.insight.181096DS1), including markers for different immune cell populations and markers for activation and functionality. For these analyses we used a selected patient cohort where all patients had at least 2 samples taken during the disease course: a sample taken at diagnosis and at relapse (21 patients/45 samples; long-OS = 11/23, short-OS = 10/22) (Supplemental Table 2 and Figure 2). Time points of relapse sampling are noted in Supplemental Table 3, and the gating strategy is shown in Supplemental Figure 1. We also included age-matched healthy control (HC) samples in the analysis.

### Increased percentage of monocytes and decreased percentage of T cells in patients with short survival.

**Figure 3:**
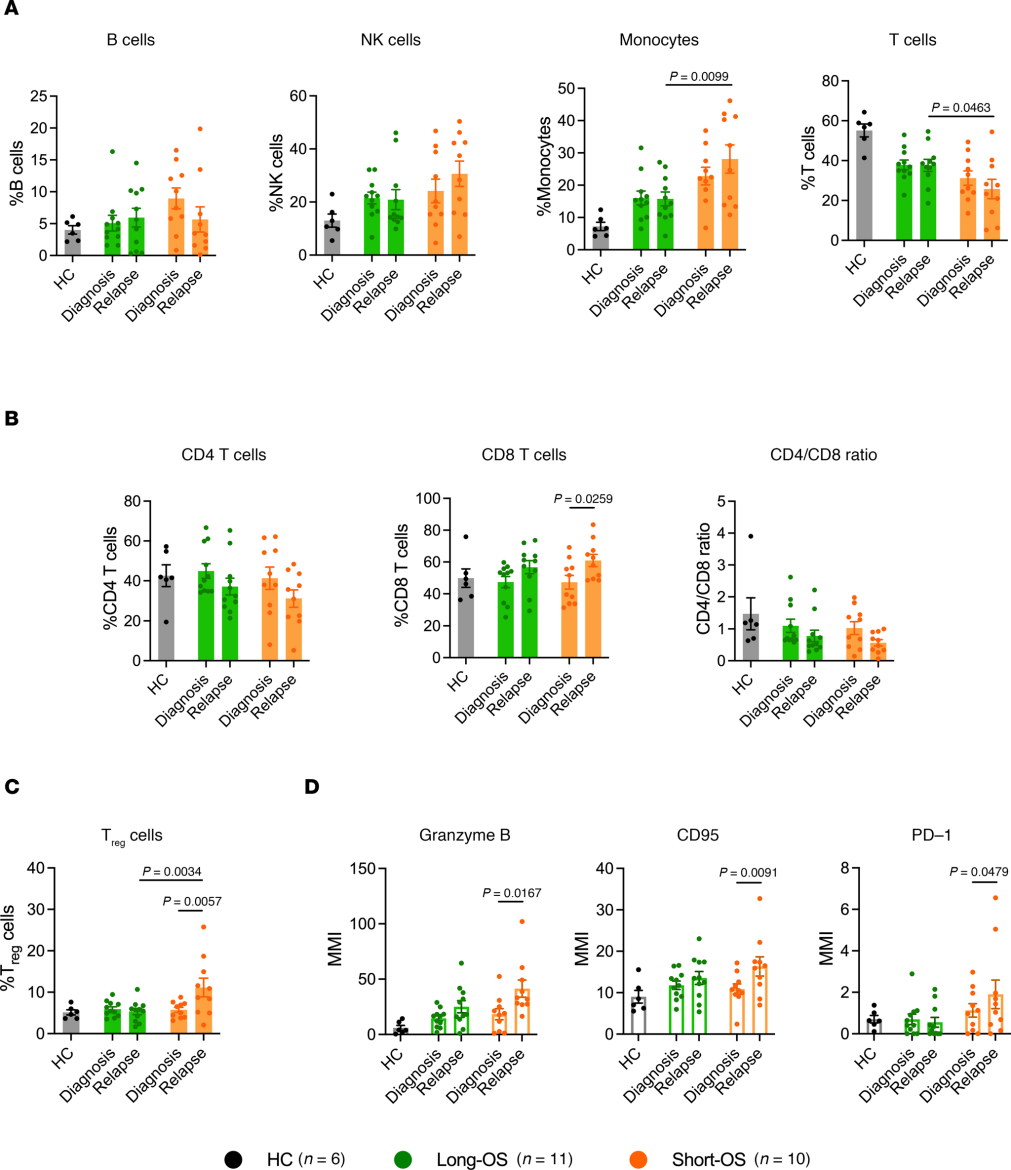
Mass cytometry analysis of B cells, NK cells, monocytes, T cells, and Tregs and CD8^+^ T cell expression of activation markers. Percentage of (**A**) B cells (CD138^–^CD11c^–^CD56^–^CD14^–^CD3^–^CD20^+^), NK cells (CD138^–^CD3^–^CD20^–^CD56^+^), monocytes (CD138^–^CD3^–^CD20^–^CD14^+^), and T cells (CD138^–^CD11c^–^CD56^–^CD14^–^CD20^–^CD3^+^), within the live CD45^+^ population; (**B**) CD4^+^ and CD8^+^ T cells within the CD3^+^ T cell population, as well as CD4/CD8 ratio; (**C**) Tregs (CD4^+^CD25^+^FoxP3^+^) within the CD4^+^ T cell population; and (**D**) MMI of granzyme B, CD95, and PD-1 within the CD8^+^ T cell population in the BM of patients with myeloma and age-matched HCs (*n* = 6). Paired diagnosis and last relapse samples from *n* = 11 long-OS patients and *n* = 10 short-OS patients were included. Mean ± SEM shown. Statistical significance was determined by repeated measures 2-way ANOVA and Šidák’s multiple comparisons test on long-OS and short-OS patient samples. Data from HCs are also shown. *P* values are indicated where *P* < 0.05.

Mass cytometry analysis revealed no significant changes in the B cell (CD3^–^CD20^+^) and NK cell (CD3^–^CD20^–^CD56^+^) compartments, across patient groups and sample time points (Figure 3A). Percentage of monocytes (CD3^–^CD20^–^CD14^+^), previously found to decrease in the BM of patients with myeloma relative to healthy individuals (10), was elevated in short-OS patients compared with long-OS patients at relapse (Figure 3A). These were predominantly HLA-DR^+^ (Supplemental Figure 2A). There were no significant changes in HLA-DR^–^ monocytes (Supplemental Figure 2B), which have been described as immunosuppressive MDSCs (11). T cells are of high importance in the antitumor immune response, and cytotoxic CD8^+^ T cells specifically are essential for efficient tumor cell killing. In our patient cohort, short-OS patients had a lower percentage of T cells (CD3^+^CD20^–^) within the live CD45^+^ compartment at relapse compared with long-OS patients (Figure 3A). In addition, both patient groups showed a trend toward decrease in CD4^+^ T cells and increase in CD8^+^ T cells, the latter being significant in short-OS patients, mirrored by the decreasing CD4/CD8 ratio from diagnosis to relapse (Figure 3B).

### Increased percentage of Tregs and elevated expression of activation markers in patients with short survival.

To investigate functionality and activation of the T cell compartment, we analyzed percentage of Tregs (CD4^+^CD25^+^FoxP3^+^), as well as expression and median mass intensity (MMI) of the markers CD95 (Fas), granzyme B, and programmed cell death 1 (PD-1) in the CD8^+^ T cell population. Expression of CD95 and granzyme B is associated with CD8^+^ T cell activation and tumor cell killing, whereas activation of the programmed cell death ligand 1 (PD-L1)/PD-1 pathway leads to inhibition of T cell activation (12). While these measures of functionality and activation remained relatively stable in long-OS patients, we saw an increase in percentage of Tregs (Figure 3C) and CD8^+^ T cell expression of CD95, granzyme B, and PD-1 (Figure 3D) in short-OS patients at relapse, suggesting a less stable T cell environment.

### Activation of naive CD8^+^ T cells in patients with short survival.

**Figure 4:**
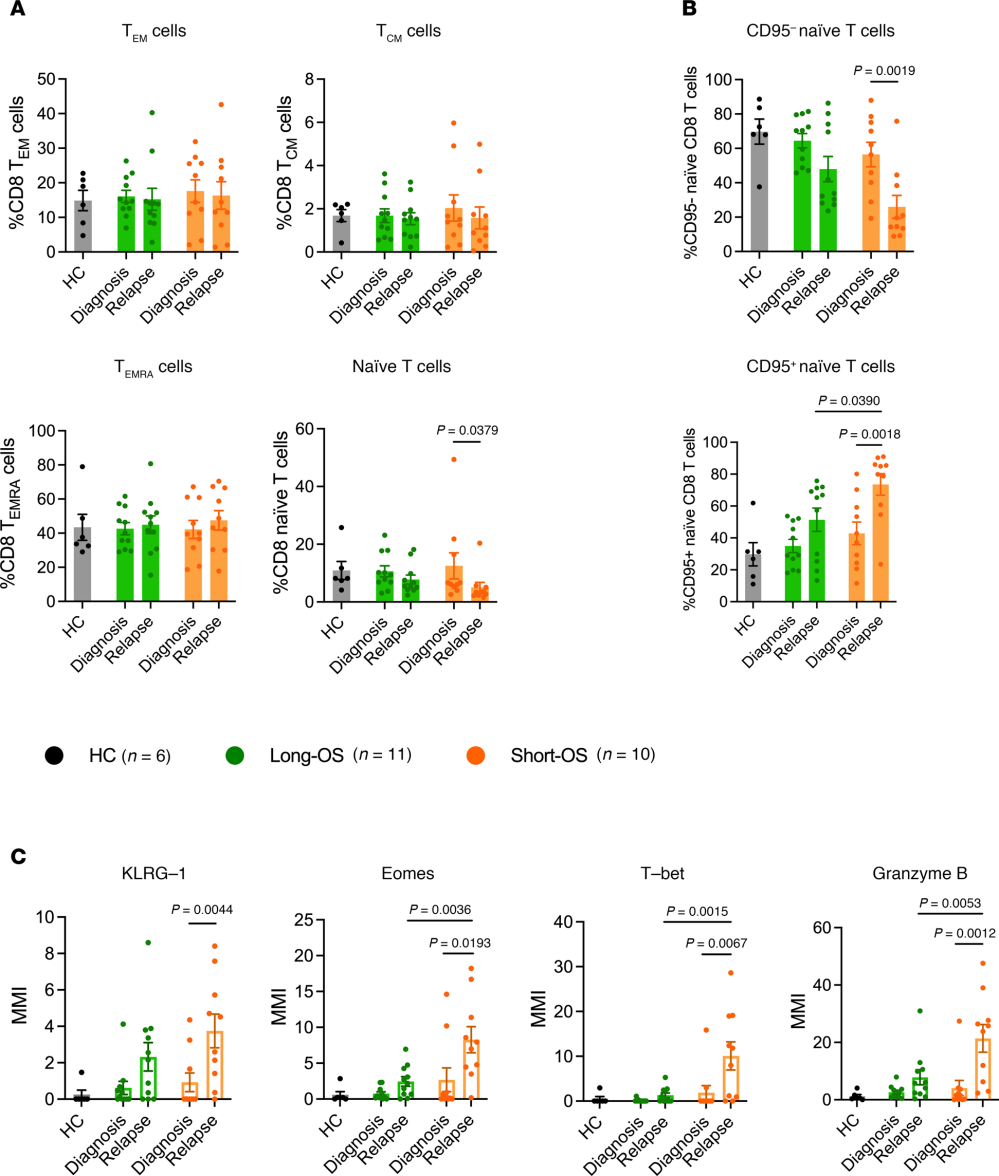
Mass cytometry analysis of naive, memory, and effector CD8^+^ T cell populations. Percentage of (**A**) T_CM_ (CD45RA^–^CD45RO^+^CCR7^+^), T_EM_ (CD45RA^–^CD45RO^+^CCR7^–^), TEMRA (CD45RA^+^CD45RO^–^CCR7^–^), and naive CD8^+^ T cells (CD45RA^+^CD45RO^–^CCR7^+^) within the CD8^+^ T cell population; (**B**) CD95^–^ and CD95^+^ naive CD8^+^ T cells within the naive CD8^+^ T cell population; and (**C**) MMI of KLRG-1, Eomes, T-bet, and granzyme B within the naive CD8^+^ T cell population, in the BM of patients with myeloma and age-matched HCs (*n* = 6). Paired diagnosis and last relapse samples from *n* = 11 long-OS patients and *n* = 10 short-OS patients were included. Mean ± SEM shown. Statistical significance was determined by repeated measures 2-way ANOVA and Šidák’s multiple comparisons test on long-OS and short-OS patient samples. Data from HCs are also shown. *P* values are indicated where *P* < 0.05.

Memory T cells are more long-lived than effector T cells and have been linked to survival and response to therapy in several malignancies (13–16), while effector T cells and effector memory RA^+^ T (TEMRA) cells were increased in patients with myeloma relative to healthy individuals (7). Thus, we investigated CD8^+^ T effector and memory cell subsets in our patient cohort. Based on expression of CD45RA, CD45RO, and CCR7, we identified T effector memory (T_EM_) cells (CD45RA^–^CD45RO^+^CCR7^–^), T central memory (T_CM_) cells (CD45RA^–^CD45RO^+^CCR7^+^), TEMRA (CD45RA^+^CD45RO^–^CCR7^–^) cells, and naive T cells (CD45RA^+^CD45RO^–^CCR7^+^) (17). This analysis revealed a decrease of naive CD8^+^ T cells at relapse for short-OS patients and stability in the T_EM_, T_CM_, and TEMRA cell populations (Figure 4A). As ASCT affects the frequency of naive T cells (18), patients were subgrouped according to whether they received ASCT (Supplemental Table 3) and reanalyzed, revealing no difference between treatment groups (data not shown).

Within the naive CD8^+^ T cell population, CD95 expression has been used to define CD95^–^ naive T cells and CD95^+^ stem cell memory T (T_SCM_) cells, the latter being characterized by sustained self-renewal potential and associated with superior antitumor immunity (19–21). Thus, we determined the proportions of CD95^+^ and CD95^–^ cells within the naive CD8^+^ T cell subset and found a significant shift from CD95^–^ naive CD8^+^ T cells to CD95^+^CD8^+^ T_SCM_ cells in short-OS patients (Figure 4B). This suggests a shift toward a more activated phenotype, especially in short-OS patients, at relapse.

To analyze this further, we looked at expression of KLRG-1, Eomes, T-bet, and granzyme B within the naive CD8^+^ T cell population. At relapse, we found increased expression of all these markers in short-OS patients, also compared with long-OS patients at the same time point, when the expression remained relatively stable (Figure 4C), again suggesting increased activation of naive CD8^+^ T cells in short-OS patients. Upon investigating expression in patients subgrouped according to ASCT treatment history, we found generally higher expression of KLRG-1, Eomes, T-bet, and granzyme B in ASCT-treated short-OS patients at relapse, the difference in KLRG-1 expression being significant, suggesting that this change could to some extent be related to ASCT (Supplemental Figure 3).

### Increased CD8^+^ T cell exhaustion in patients with short survival.

**Figure 5:**
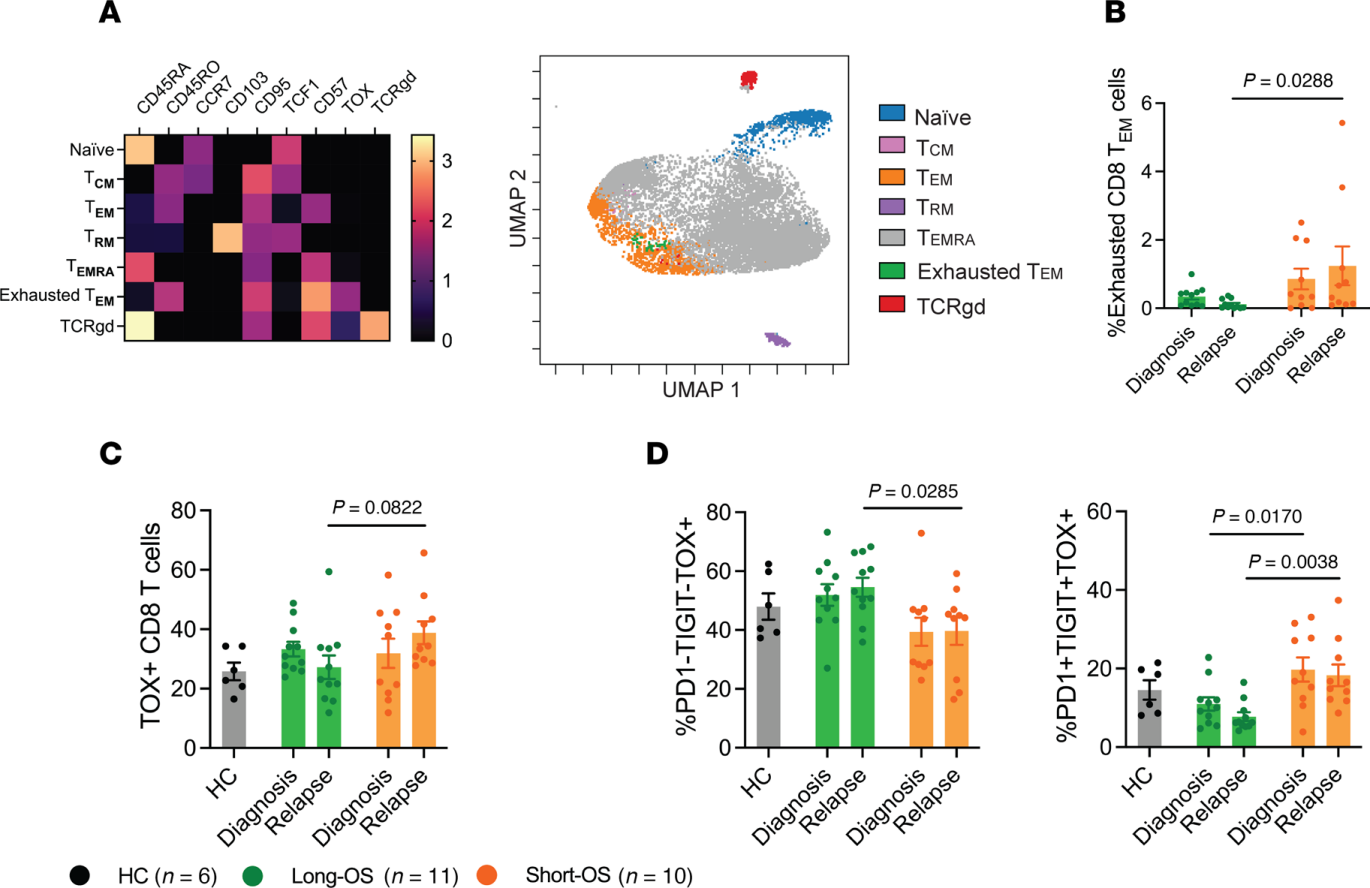
Increased CD8^+^ T cell exhaustion in myeloma patients with short survival. (**A**) Heatmap showing the transformed ratio by column’s minimum MMI of selected markers for 7 populations (FlowSOM clustering using equal sampling and hierarchical consensus clustering) (left) and overlaid uniform manifold approximation and projection (UMAP) of the clusters found by FlowSOM analysis (right). TOX, thymocyte selection-associated high mobility group box protein; TIGIT, T cell immunoreceptor with immunoglobulin and ITIM domains. (**B**) Percentage of exhausted CD8^+^ T_EM_ cells, as found by FlowSOM clustering, within the CD8^+^ T cell population. (**C**) Percentage of TOX^+^CD8^+^ T cells within the CD8^+^ T cell population, including age-matched HCs (*n* = 6), and (**D**) percentage of PD-1^–^TIGIT^–^TOX^+^CD8^+^ T cells (left) and PD-1^+^TIGIT^+^TOX^+^CD8^+^ T cells (right) within the TOX^+^CD8^+^ T cell population, as found by manual gating. Diagnosis and all available relapse samples were used for FlowSOM clustering. Heatmap and UMAP shown for a representative experiment. Paired diagnosis and last relapse samples from *n* = 11 long-OS patients and *n* = 10 short-OS patients are included in the bar graph. Mean ± SEM shown. Statistical significance was determined by repeated measures 2-way ANOVA and Šidák’s multiple comparisons test on long-OS and short-OS patient samples. Data from HCs are also shown. *P* values are indicated where *P* < 0.05.

To investigate the CD8^+^ T cell compartment with an unbiased method, we performed FlowSOM (22) analysis, an unsupervised machine learning clustering technique. In the CD8^+^ T cell population, FlowSOM analysis identified 7 distinct naive, memory, effector, and exhausted T cell populations (Figure 5A) and, as with manual gating, showed significant decrease of naive CD8^+^ T cells for short-OS patients (Supplemental Figure 4). FlowSOM analysis also revealed a cluster expressing high levels of TOX, which is highly involved in T cell exhaustion (23), and this cluster was defined as a subpopulation of exhausted CD8^+^ T_EM_ cells. At relapse, this population was significantly higher in short-OS patients than long-OS patients (Figure 5B), suggesting that the CD8^+^ T_EM_ cells of short-OS patients are more exhausted at relapse than those of long-OS patients. Using manual gating, we found a trend of increased TOX^+^CD8^+^ T cells in short-OS patients at relapse (Figure 5C), although this was not significant. It has been demonstrated that T cell exhaustion is progressive, with cumulative increase in diversity and amount of inhibitory receptors expressed (24, 25). To explore this, we analyzed expression of PD-1 and TIGIT within the TOX^+^CD8^+^ T cell population. Interestingly, we found that long-OS patients had more PD-1^–^TIGIT^–^TOX^+^CD8^+^ T cells and fewer PD-1^+^TIGIT^+^ TOX^+^CD8^+^ T cells overall. Short-OS patients had significantly fewer PD-1^–^TIGIT^–^TOX^+^CD8^+^ T cells and more PD-1^+^TIGIT^+^TOX^+^CD8^+^ T cells than long-OS patients at relapse, the latter being significant also at diagnosis (Figure 5D). This suggests that the CD8^+^ T cells in short-OS patients are more exhausted and less functional than in long-OS patients. Data from age-matched HCs showed similar trends to what was observed in long-OS patients (Figures 3–5).

### PD-1 expression may distinguish patients with long and short survival at diagnosis.

**Figure 6:**
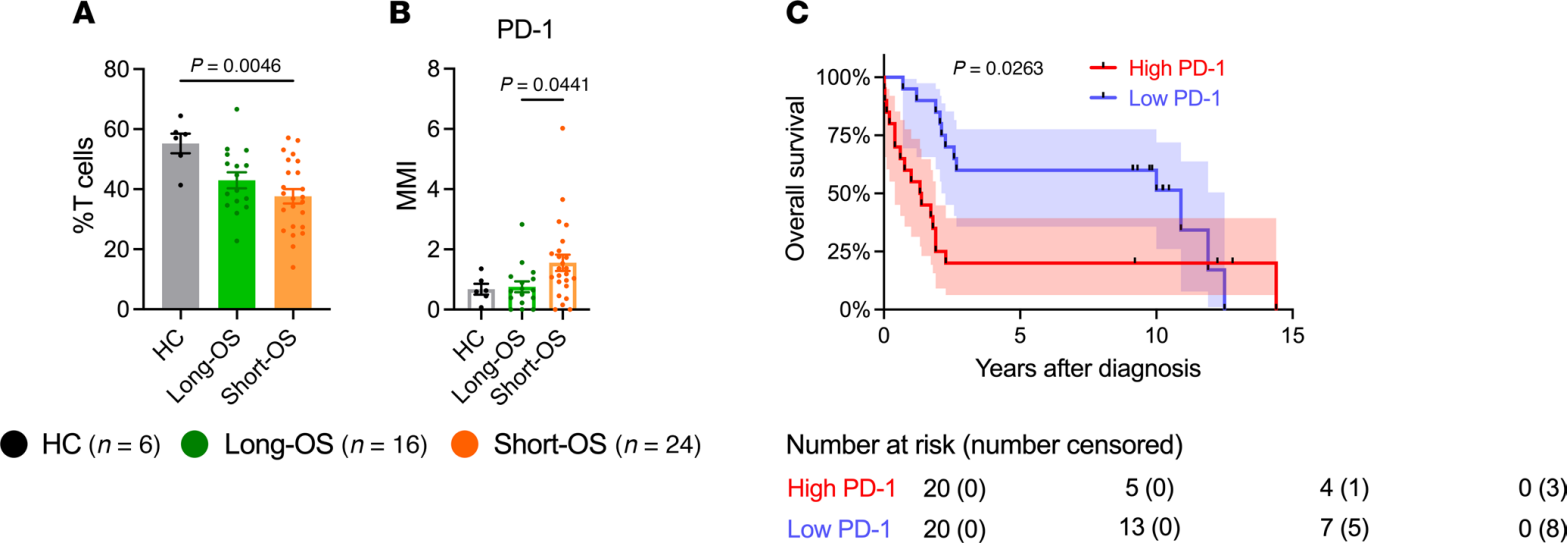
Lower percentage of T cells and increased PD-1 expression of CD8^+^ T cells at diagnosis in myeloma patients with short survival and low PD-1 expression associated with OS. (**A**) Percentage of T cells within the live CD45^+^ population, and (**B**) MMI of PD-1 within the CD8^+^ T cell population, in the BM of HCs and patients with myeloma at diagnosis. (**C**) Kaplan-Meier survival analysis of OS from diagnosis for all patients stratified according to high or low PD-1 MMI (above or below median = 1.03) within the CD8^+^ T cell population at diagnosis. Samples from *n* = 6 HCs, *n* = 16 long-OS patients, and *n* = 24 short-OS patients at diagnosis were included. Mean ± SEM shown. Statistical significance was determined by Kruskal-Wallis test and Dunn’s multiple comparisons test (**A** and **B**) or log-rank test (**C**). *P* values are indicated where *P* < 0.05.

To identify markers that could be used to predict survival, we analyzed an expanded patient cohort where we included 19 additional patients for which we had only a diagnosis sample available, giving a total of 40 patients (long-OS = 16, short-OS = 24) (Figure 2 and Supplemental Figure 5), as well as 6 age-matched HCs. When comparing samples taken at diagnosis, our analysis showed few differences in percentage of immune populations or expression of activation markers with our panel of antibodies. There was a significantly lower percentage of T cells in short-OS patients relative to HCs (Figure 6A), but no significant changes in CD8^+^ or CD4^+^ T cells were found (Supplemental Figure 6A). Interestingly, when investigating activation markers, we found significantly higher PD-1 expression within the CD8^+^ T cell population in short-OS patients compared with long-OS patients at diagnosis (Figure 6B). Moreover, when dividing patients into groups of high or low PD-1 expression, Kaplan-Meier survival analysis of OS revealed that low PD-1 expression was significantly associated with OS in our patient cohort (Figure 6C), suggesting PD-1 expression in CD8^+^ T cells may distinguish long-OS and short-OS patients at diagnosis. However, multivariate analysis taking ISS and age into consideration failed to retain a significant correlation (*P* = 0.197, Table 2). No significant changes in percentage of Tregs or CD95 or granzyme B expression were found (Supplemental Figure 6, B and C), and no significant differences in naive CD8^+^ T cells or CD95^+^/^–^ cells within the naive population could be detected at diagnosis (Supplemental Figure 7).

### Increase of hyperexpanded clones in patients with short survival.

To explore the T cell repertoire and clonality, we performed TCR-Seq on a subset of patients (total = 13 patients/26 samples, long-OS = 8/16, short-OS=5/10) (Figure 2 and Supplemental Table 2). We analyzed paired samples from either diagnosis or early progression (“early” sample) and later progression (“late” sample) to explore changes in the T cell repertoire during the disease course in the 2 patient groups. TCR repertoire metrics are given in Supplemental Table 2.

**Figure 7:**
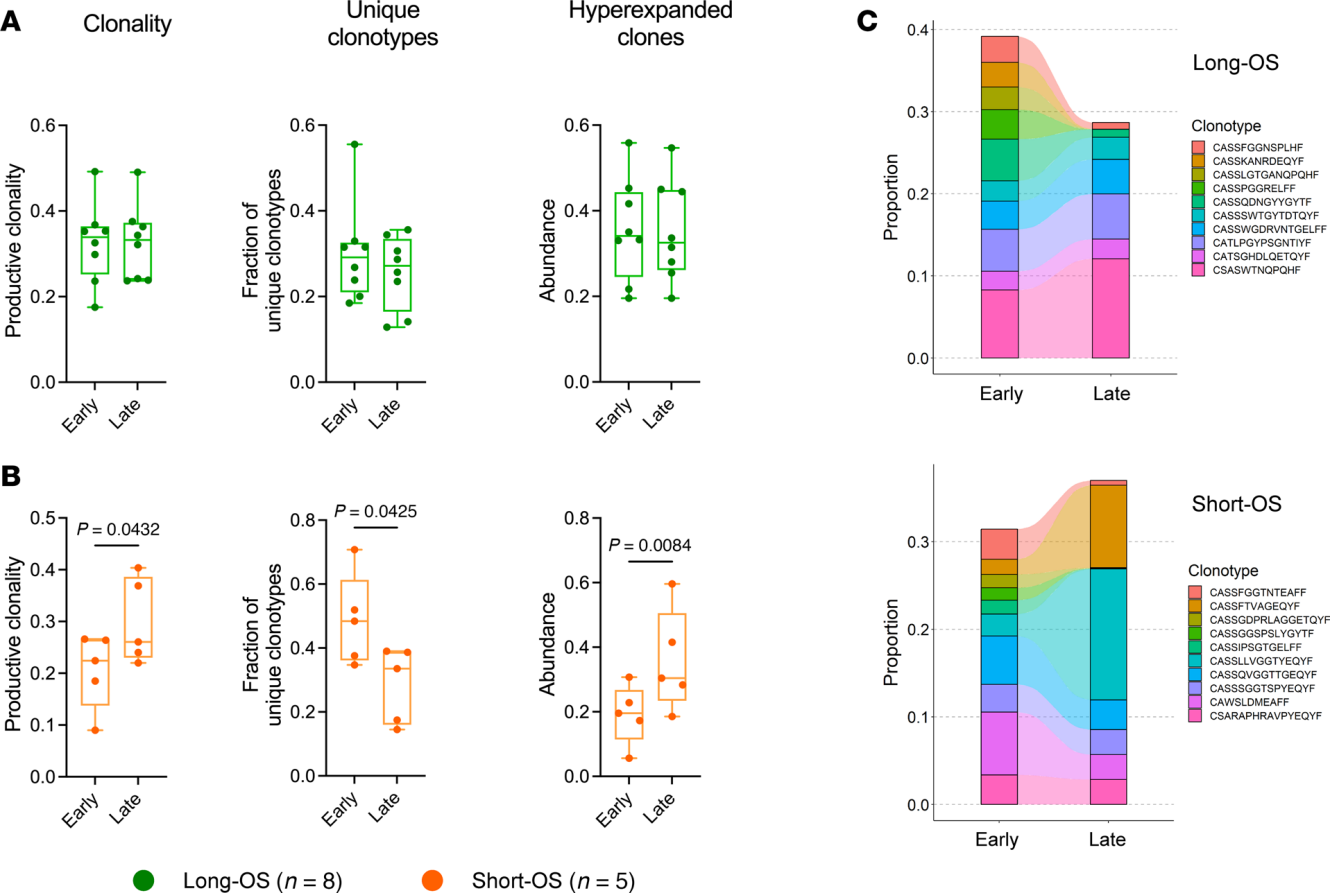
Bulk TCR-Seq of BM reveals increase in clonality and abundance of hyperexpanded clones in myeloma patients with short survival. (**A** and **B**) Box plots showing productive clonality, fraction of unique clonotypes, and abundance of hyperexpanded clones at early and late BM sampling of short-OS (**A**) and long-OS (**B**) patients. (**C**) Clonotype tracking of the top 10 clonotypes at early sampling for 1 representative short-OS patient (top) and 1 representative long-OS patient (bottom). Paired early (diagnosis or relapse) and late (latest relapse available) samples from *n* = 8 long-OS patients and *n* = 5 short-OS patients were included. Box plot center represents the median, bounds of the box represent the first and third quartiles, and whiskers indicate full range of values. Statistical significance was determined by paired *t* test. Shapiro-Wilk test was used to evaluate normality of the data. *P* values are indicated where *P* < 0.05.

Productive clonality, fraction of unique clonotypes, and abundance of hyperexpanded clones remained relatively stable in long-OS patients (Figure 7A). In short-OS patients, however, we observed a significant (*P* = 0.0432) increase in productive clonality, with a concurrent decrease in fraction of unique clonotypes (*P* = 0.0425) from early to late sampling. Furthermore, the abundance of hyperexpanded clones increased significantly (*P* = 0.0084) for short-OS patients (Figure 7B). There were no significant differences in age between the short-OS and long-OS patients in this cohort (Supplemental Table 4), and when accounting for age, using multivariate analysis in SPSS (Supplemental Table 5), the significant *P* values for the increase in hyperexpanded clones and decrease in unique clonotypes remained. However, in contrast with what was seen using paired 2-tailed *t* test, multivariate analysis using the difference between early and late time points for each patient, the increase in productive clonality was no longer significant (ANOVA *P* = 0.062) (Figure 7B; *P* = 0.0432), suggesting that these findings should be verified in a larger patient cohort. By performing clonotype tracking, we also found that the distribution of the top 10 clones was relatively stable from early to late disease in long-OS patients, whereas in short-OS patients, we clearly observed expansion of some clonotypes, and diminishing of others, mirroring the increase in productive clonality and abundance of hyperexpanded clones (Figure 7C). Moreover, grouping of lymphocyte interactions by paratope hotspots, version 2 (GLIPH2) (26), amino acid sequence motif analysis revealed 3 recurrent complementarity-determining region 3 (CDR3β) sequence motifs among the most abundant T cell clones from short-OS patients, where sequences from 4/5 patients were represented in each group (index 1, 3, and 4) (Supplemental Table 6), suggesting increased likelihood of shared antigen specificity in short-OS patients. Taken together, this indicates a shift in the T cell environment of short-OS patients, from a more polyclonal T cell repertoire, toward an environment richer in hyperexpanded, monoclonal T cells, while the T cell repertoire of long-OS patients appears to be more stable.

## Discussion

In this study, we compared the TME of myeloma patients with long and short OS, both at the time of diagnosis and at relapse. This patient cohort with sequential samples provides insight into the development of the TME from diagnosis to relapse and how this might relate to survival and disease control. While we could not detect changes in long-OS patients, our data illustrate several changes in the T cell environment in short-OS patients. Our results show that short-OS patients at relapse have fewer T cells and more Tregs, CD8^+^ T cells with a more activated and exhausted phenotype, and a more monoclonal TCR repertoire with higher abundance of hyperexpanded clones.

Previous studies found increased expression of markers associated with T cell exhaustion, such as TOX, PD-1, and cytotoxic T lymphocyte antigen 4 (CTLA-4), in the BM of both newly diagnosed and treated patients with myeloma when compared with healthy donors (7, 27–29), and an increase in Tregs has been linked to adverse clinical features and elevated risk of progression (30). Our data indicate that increase in PD-1 expression and percentage of Tregs and exhausted T cells is associated with short-OS. Exhaustion of T cells in the TME, first demonstrated in melanoma in 1999 (31), is caused by chronic stimulation of T cells by tumor antigens and inhibitory ligands, such as PD-L1 (32). As PD-L1 is highly expressed on most PCs of patients with myeloma (33, 34), it would be reasonable to expect PD-L1 expression in our cohort as well. Currently, we have data on tumor PD-L1 expression in 3 of these patients from previous RNA-Seq data (35), where 2 of 3 show PD-L1 expression (transcripts per million [TPM] > 1). Increase in PD-1 expression in the CD8^+^ T cell population suggests that the PD-L1/PD-1 pathway could contribute to exhaustion and inhibition of function in the CD8^+^ T cell compartment in these patients. We also found that short-OS patients had significantly more PD-1^+^TIGIT^+^ double-positive cells within the TOX^+^CD8^+^ T cell population, compared with long-OS patients, who had significantly more PD-1^–^TIGIT^–^ double-negative cells, suggesting that the CD8^+^ T cells in short-OS patients are progressively more exhausted than the CD8^+^ T cells in long-OS patients (24, 25). It has been demonstrated that T cell exhaustion is progressive during chronic viral infection (24), and TOX expression has been found within polyfunctional T_EM_ cells as well as exhausted T cells (36). Other checkpoint molecules, such as CTLA-4 and VISTA (37, 38), could be involved as well, contributing to the exhausted TOX^+^ phenotype, but these markers were not included in our panel.

Naive CD8^+^ T cells are central components of the T cell response, giving rise to memory and effector CD8^+^ T cell subsets, and have, for instance, been associated with higher likelihood of robust clinical response to chimeric antigen receptor T B- cell maturation antigen therapy (39). In this study, we found a significant decrease of naive CD8^+^ T cells at relapse in short-OS patients (Figure 4A), which could negatively affect antitumor responses and response to treatment. By analyzing this population further, we found higher expression of activation markers and a shift toward a more activated CD95^+^ T_SCM_ cell phenotype. T_SCM_ cells are generally regarded as a beneficial, self-renewing population with increased proliferative capacity, and generation of T_SCM_ cells has been found to be important for diversification of immunological memory after hematopoietic stem cell transplantation (40). Furthermore, T_SCM_ cells have been shown to produce inflammatory cytokines, including IFN-γ, IL-2, and TNF-α (19), which can contribute to the activation of T cells and a successful immune response. However, one could hypothesize that it may contribute to the pro-inflammatory TME and lead to a dysfunctional tumor response in short-OS patients. Activation of T cells is necessary to eliminate malignant cells, but dysregulation of the signals involved can lead to an imbalance that impairs the function and efficiency of the T cell response. Granzymes, for instance, are very important in the immune response but can also contribute to development and progression of pathologies if they are dysregulated (41).

There are several clinical parameters in our cohort of patients that could also be influencing immune composition, for instance, rate of infections. Patients with myeloma have increased rates of infection, which has been linked to both the immunodeficient nature of the disease impairing the immune system, as well as the different treatments affecting the balance and functions of the TME (42, 43). Unfortunately, we do not have a complete overview of the rate of infections in our patient cohort. Moreover, patients in our cohort did not receive the same course of treatment, which could also affect composition of the TME. For instance, ASCT could lead to activation of naive T cells, which may contribute to the increase seen in CD95^+^ naive T cells. It should also be noted that, because of the retrospective nature of this study, treatments administered to our cohort of patients do not fully reflect today’s standard of care. Daratumumab-based combinations, for instance, are a common treatment today (44, 45), but only 3 patients in our cohort received them. Another clinical parameter that is known to influence immune composition is tumor burden. Higher percentage of PCs has been linked to, for instance, T cell exhaustion and reduced treatment efficacy (46–48). In our cohort, median percentage of PCs was consistently higher for short-OS patients, with the only exception being in the TCR-Seq cohort at last sampling, where the opposite was observed (Table 1 and Supplemental Table 4). This could indicate that higher PC percentages may correlate with poorer outcomes in our cohort, though the exception in the TCR-Seq cohort highlights the need to further explore these dynamics over time.

Low PD-1 expression on CD8^+^ T cells at diagnosis was associated with improved survival in a univariate analysis; however, whether that can be used on its own to predict survival is unlikely, as multivariate analysis taking ISS or age into consideration did not show a significant correlation. However, in line with our data, an earlier study investigating biopsies from a larger patient cohort found that high PD-1 expression on T cells at diagnosis was associated with inferior OS independent of ISS (49).

Results from TCR-Seq also illustrate a less stable TME in short-OS patients over time, with an increase in clonality and abundance of hyperexpanded clones, and a concurrent decrease in the fraction of unique clonotypes. Due to the limitations of bulk sequencing, we can only speculate which subpopulations of T cells are expanding and contracting, but with the increase in activation and exhaustion found by mass cytometry in mind, one could hypothesize that the expanding TCR clones might have higher expression of activation markers or even be of a Treg or exhausted phenotype. In melanoma, single-cell TCR-Seq has demonstrated that the highest level of clonal expansion is displayed by dysfunctional T cells (50), and in myeloma large (hyperexpanded) T cell clones have been found to express multiple immune checkpoint proteins and possibly predict resistance to IMiD therapy (46). Another study found enrichment of dysfunctional T cells in patients with suboptimal response to bortezomib-melphalan-prednisolone treatment (51). It can be speculated that the hyperexpanded T cell clones in short-OS patients might be dysfunctional and contribute to impaired treatment responses. In contrast, the hyperexpanded clones in long-OS patients might be more cytotoxic or even tumor-specific. However, further research is needed to confirm these hypotheses. Another interesting aspect for investigation is whether these expanded T cell clones are specific for tumor antigen. GLIPH2 analysis suggests that the most expanded clones in short-OS patients might share antigen specificity, and something similar has been reported in myeloma with unsustained minimal residual disease negativity (52), but finding what exactly they are specific for requires further investigation. Finally, to overcome the limitations of bulk TCR-Seq and investigate the phenotype of the expanded T cell clones further, it would be of great interest to expand to other methods, such as ATAC, RNA, or TCR single-cell sequencing.

Noting that the sample size is relatively small and noting the heterogeneous treatment, we can conclude that these findings indicate significant changes over time in the TME of short-OS myeloma patients, specifically in the T cell compartment, with a decrease in overall T cells, an increase in immunosuppressive Treg cells, and a shift toward a more activated and exhausted phenotype in the CD8^+^ T cell compartment. Additionally, abundance of hyperexpanded T cell clones in short-OS patients warrants further investigation of the phenotype, functionality, activation, and antigen specificity of these clones and the T cell repertoire as a whole.

## Methods

### Sex as a biological variable.

Our study examined male and female patients/HCs, and similar findings are reported for both sexes.

### Patients and samples.

Patients with MM (*N* = 45), from Biobank1 (St. Olavs Hospital, Trondheim, Norway) (*n* = 17), Finnish Hematology Registry and Clinical Biobank (FHRB) Biobank (Helsinki, Finland) (*n* = 8), Aalborg Biobank (Aalborg University Hospital, Aalborg, Denmark) (*n* = 8), and KI Biobank (Karolinska Institutet, Stockholm, Sweden) (*n* = 12), were selected based on criteria related to OS. Patients with more than 8 years between diagnosis and death (OS > 8 years) were termed long-OS patients (*n* = 21) and those with fewer than 3 years between diagnosis and death (OS < 3 years) short-OS patients (*n* = 24). For 26 patients (long-OS = 16, short-OS = 10), 2 or more sequential BM samples from each patient were available, while 19 patients had a sample taken at diagnosis only (long-OS = 5, short-OS = 14). Between diagnosis and last available sequential sample analyzed, long-OS patients received a median of 2 lines of therapy (range: 1–7), while short-OS patients received a median of 1 line of therapy (range: 1–8). Therapies administered more than once were counted as a single line, and radiotherapy was excluded from the count. Two patients were excluded from the calculation of median lines of therapy because of incomplete treatment histories (Supplemental Table 7). BM samples from age-matched HCs were also included (Biobank1) (*n* = 6). Thawed CD138-negative cells (isolated from fresh BMMNCs by RoboSep automated cell separator and Human CD138 Positive Selection Kit; 17877RF, STEMCELL Technologies) from Norwegian and Swedish cohorts and BMMNCs from the Finnish and Danish cohorts were used for the analysis. The patients received treatment in accordance with clinical routines for myeloma and were not enrolled in clinical studies. Therefore, therapy was not standardized across individuals or groups. The median age at the time of diagnosis for included patients was 61 years (range 38–81), 71 years (range 42–87), and 56 years (range 53–65) for long-OS patients, short-OS patients, and HCs, respectively. Clinical data for patients with sequential samples and patients with diagnosis samples only are summarized in Supplemental Tables 3 and 8, and treatment history for the former group is given in Supplemental Table 7.

### Mass cytometry.

Thawed suspensions of CD138-negative BMMNCs or BMMNCs were stained using a custom 37-marker panel with metal-conjugated antibodies (Supplemental Table 1), both preconjugated (Standard BioTools) and conjugated in-house using a conjugation kit (Standard BioTools). Cells were stained, permeabilized, fixed, and washed as specified by the manufacturer’s cell surface and nuclear antigen staining protocols (Standard BioTools). After antibody staining, cells were frozen at –80°C until analysis. Samples were then thawed, stained with intercalation solution (Standard BioTools), and analyzed using a Helios or XT mass cytometer (Standard BioTools).

### Genomic DNA isolation.

Vials of CD138-negative BMMNCs (10 million cells/vial) stored in nitrogen were thawed in a 37°C water bath. Prewarmed thawing medium consisting of RPMI-1640 (+glutamine; Sigma-Aldrich) with 10% FCS and 20 μg/mL DNase (Roche) was added dropwise to the cells before transferring the cells to a new tube. Cells were left at room temperature for 10–15 minutes before spinning down at 400*g* for 8 minutes. Samples were carefully aspirated and resuspended in 600 μL cold Dulbecco’s PBS before transferring to 1.5 mL Eppendorf tubes. Each sample was split into 2 aliquots, isolating DNA from one and storing the other at –80°C for future use. The samples were spun down at 0.8*g* for 6 minutes, then aspirated, and DNA was isolated using the AllPrep Mini DNA/RNA and QIAmp DNA Mini kits (QIAGEN), in accordance with the manufacturer’s protocols. Genomic DNA concentration and purity were determined using a DS-11+ spectrophotometer (DeNovix).

### Immunosequencing.

For TCR-Seq, genomic DNA was isolated from CD138-negative BMMNCs, and either prepared using the ImmunoSEQ hsTCRB v3 Kit (Adaptive Biotechnologies), followed by sequencing on a MiSeq V3 (Illumina) using the 150-cycle kit at the Genomic Core Facility (Norwegian University of Science and Technology), or shipped to ImmunoSEQ (Adaptive Biotechnologies), where the samples were prepared and sequenced using the ImmunoSEQ hsTCRBv4 kit. The amount of DNA and number of samples on each flow cell were adjusted to achieve a deep sequencing, following the manufacturer’s protocol.

### Data analysis.

Mass cytometry data were analyzed using Cytobank (Beckman Coulter). Gaussian gating was performed for data cleanup, and dead cells and doublets were excluded using cisplatin and DNA intercalators, respectively. FlowSOM (22) and UMAP (53) analyses were performed using the Cytobank software. Preliminary analysis, summary statistics, and quality control of CDR3 sequences (templates) were performed using the online ImmunoSEQ Analyzer portal (Adaptive Biotechnologies, http://www.immunoseq.com). Processed sequencing data were exported from ImmunoSEQ Analyzer and analyzed in R (version 4.3.1) using the immunarch package (54). To combine data from both kits used for sequencing, the truncated version of nucleic acid sequences was exported from ImmunoSEQ Analyzer, and for further analyses, frequency, rather than absolute count, was used to compare data. Clonality, representing the size of unique clones, was defined as 1 minus the normalized Shannon’s entropy of productive templates ranging from 0 to 1, with 0 indicating a polyclonal TCR repertoire, with no 2 clones being the same, and 1 indicating a monoclonal TCR repertoire, where all clones are the same. Hyperexpanded clones were defined as clones with a minimum frequency of 0.01 of all productive sequences. GLIPH2 was used to identify antigen specificity groups from shared CDR3 amino acid motifs within the TCRβ chain (26). Prior to this analysis, downsampling was performed to obtain an equal number of TCR reads in paired samples, followed by extraction of the top 28 expanded CDR3 sequences with a minimum frequency of 0.001.

### Statistics.

Statistical analysis of mass cytometry and TCR-Seq data was performed using GraphPad Prism 10. For mass cytometry data analysis, comparisons across long-OS and short-OS patient groups and diagnosis and relapse were made using repeated measures 2-way ANOVA and Šidák’s multiple comparisons test. For comparison between long-OS and short-OS patient groups at diagnosis and HCs, Kruskal-Wallis test and Dunn’s multiple comparisons test were used. Survival curves were estimated using the Kaplan-Meier method, and the difference between subgroups was evaluated by the log-rank test. For TCR-Seq data analysis, Shapiro-Wilk test was used to evaluate normality of the data, followed by comparison between early and late sample time points using paired 2-tailed *t* test. A significance threshold of *P* < 0.05 was used for all statistical analysis. Bar graph data were plotted as mean ± SEM, and box plots were plotted with center representing the median, bounds of the box representing the first and third quartiles, and whiskers indicating full range of values. Multivariate analysis was performed using SPSS. Cox regression analysis was performed to estimate the effect of covariates on survival. For multivariate analysis on the TCR-Seq data, the test between subject effects results from the 2-way ANOVA test in SPSS were used. For both long-OS and short-OS patient groups, the difference between late and early samples were used in the analysis.

### Study approval.

The study was approved by the Regional Committee for Medical and Health Research Ethics (REK: Mid-Norway, Trondheim, Norway) (REK 175311). Patients have given written informed consent for biobank sampling and sample use for research.

### Data availability.

Values for all data points in graphs are reported in the Supporting Data Values XLS file. Due to Norwegian law on sensitive data, sequencing data cannot be submitted to public repositories. Norwegian data protection is governed by the Law on the Processing of Personal Data (Personal Data Act) of June 15, 2018 (only available in Norwegian: https://lovdata.no/dokument/NL/lov/2018-06-15-38) (“the Act”), which implements the General Data Protection Regulation (Regulation [EU] 2016/679) (GDPR). However, all TCR-Seq data will be available upon request to the corresponding author. It will then be shared via the ImmunoSEQ Analyzer platform. A collaboration agreement will have to be signed, ensuring secure storage of the data.

## Author contributions

ADB, ENV, AMS, and KM designed the research. AW, TSS, KD, MZJ, MK, JL, VL, and AMO provided patient samples or clinical data. ADB performed research and data analysis. ADB, AMS, and KM wrote the first draft of the manuscript, and ADB, ENV, TSS, KD, MZJ, MK, JL, VL, AMO, AW, AMS, and KM reviewed the manuscript and approved its submission.

## Supplementary Material

Supplemental data

Supplemental table 1

Supplemental table 2

Supplemental table 3

Supplemental table 4

Supplemental table 5

Supplemental table 6

Supplemental table 7

Supplemental table 8

Supporting data values

## Figures and Tables

**Table 1:**
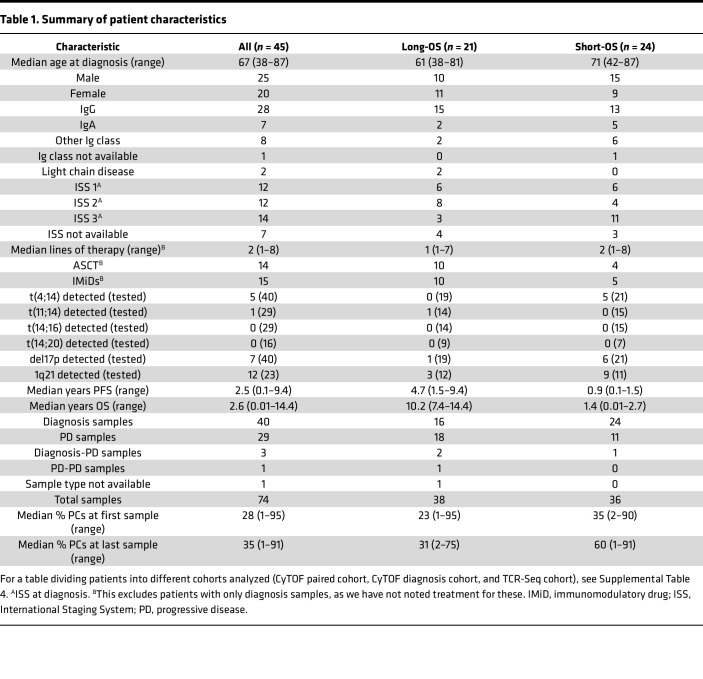
Summary of patient characteristics

**Table 2:**
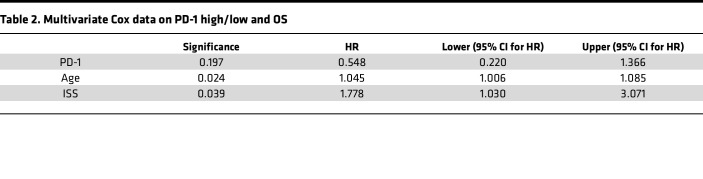
Multivariate Cox data on PD-1 high/low and OS

## Version 1 04/29/2025

In-Press Preview

## Version 2 06/09/2025

Electronic publication
