# Supplementary material for: T cell phenotype and clonality changes in myeloma patients with short overall survival
Source: JCI Insight. 2025 Apr 22;10(11):e181096. doi: 10.1172/jci.insight.181096 (PMC12220974; doi:10.1172/jci.insight.181096)
Supplement: Supplemental data [file jciinsight-10-181096-s034.pdf]

Supplemental data

A

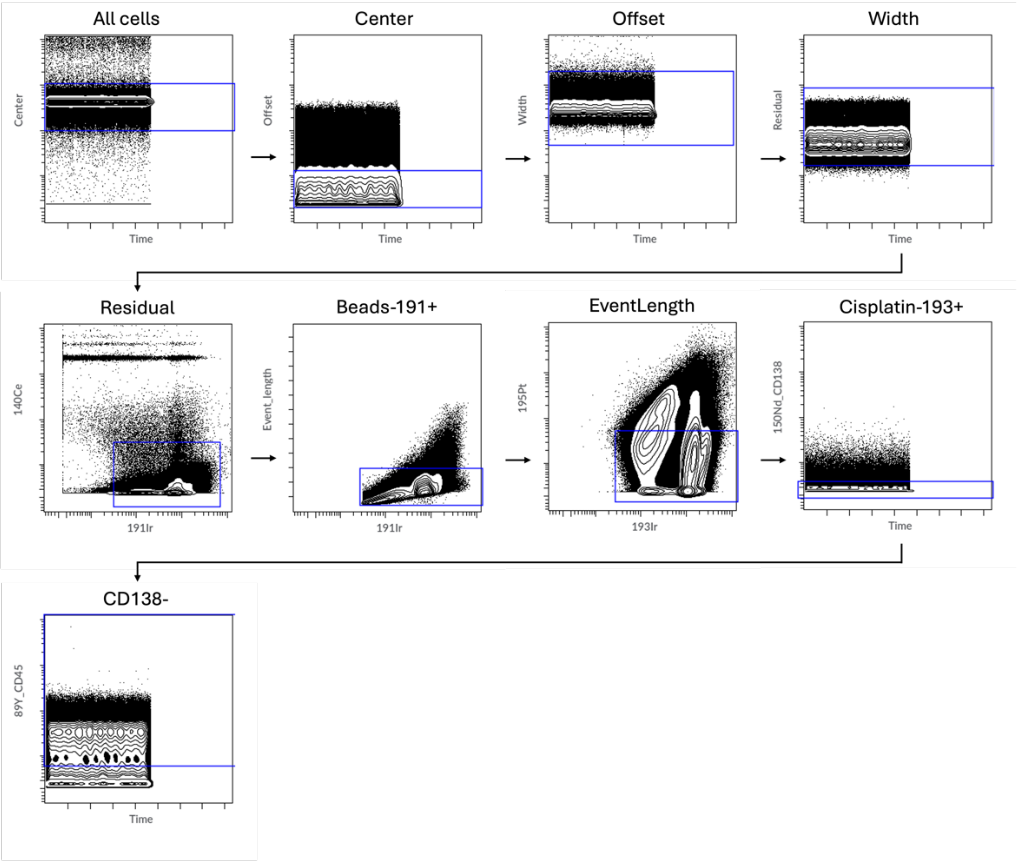

B

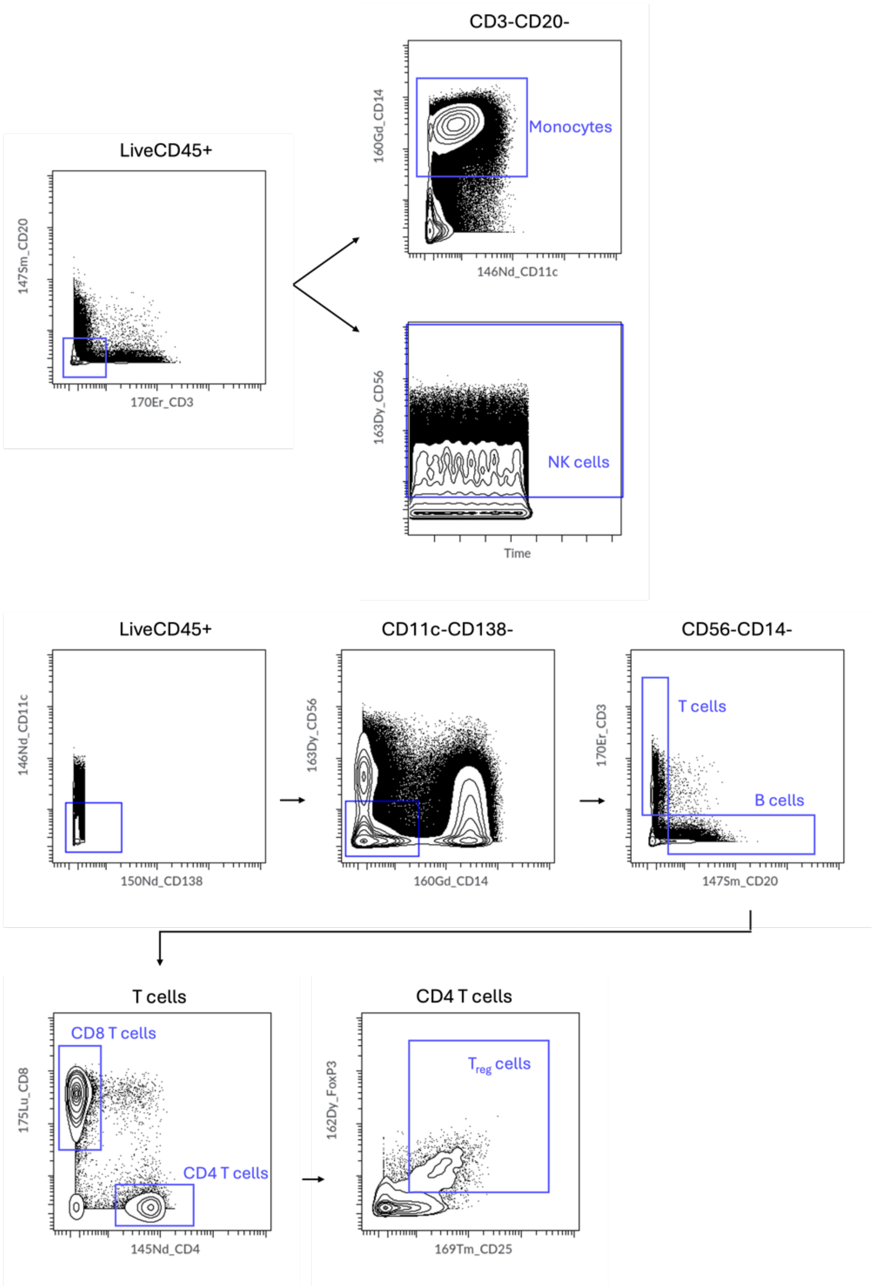

CD3-CD20-
Monocytes
160Gd_CD14
146Nd_CD11c
LiveCD45+
147Sm_CD20
170Er_CD3
NK cells
163Dy_CD56
Time
LiveCD45+
146Nd_CD11c
150Nd_CD138
CD11c-CD138-
163Dy_CD56
160Gd_CD14
CD56-CD14-
T cells
B cells
170Er_CD3
147Sm_CD20
T cells
CD8 T cells
CD4 T cells
175Lu_CD8
145Nd_CD4
CD4 T cells
T$_{reg}$ cells
162Dy_FoxP3
169Tm_CD25

C

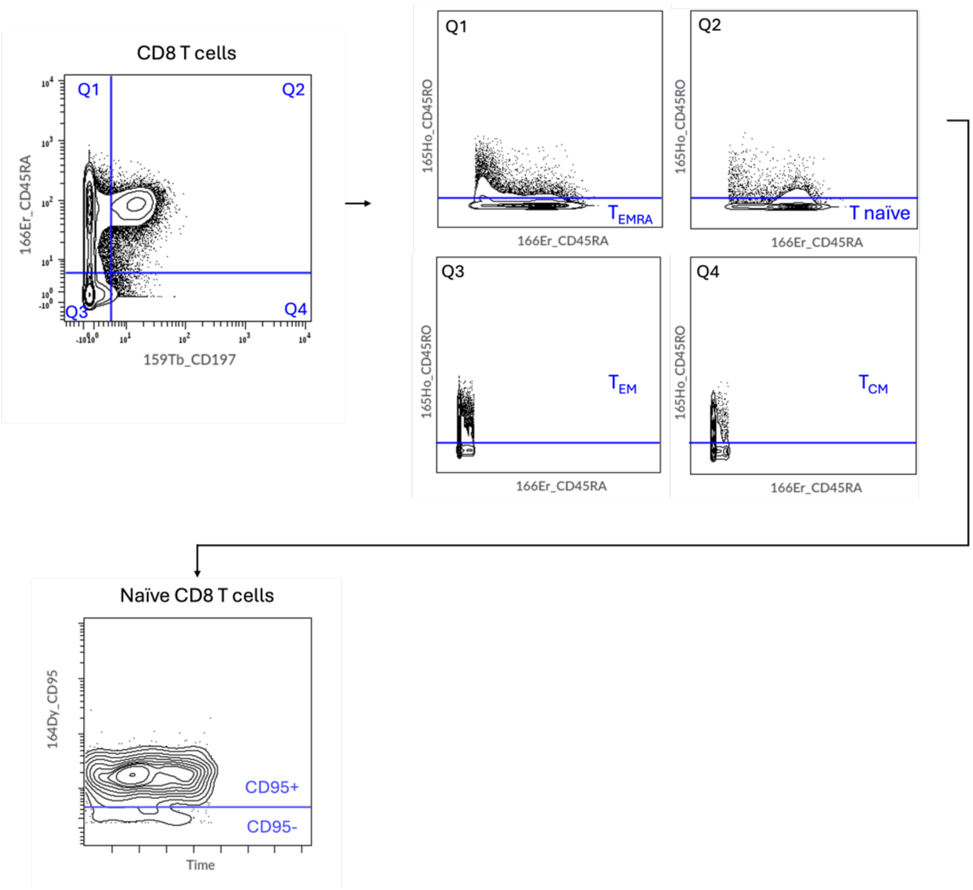
CD8 T cells
Q1
Q2
Q3
Q4
166Er_CD45RA
159Tb_CD197
165Ho_CD45RO
T EMRA
T naïve
T EM
T CM
Naïve CD8 T cells
164Dy_CD95
CD95+
CD95-
Time

D

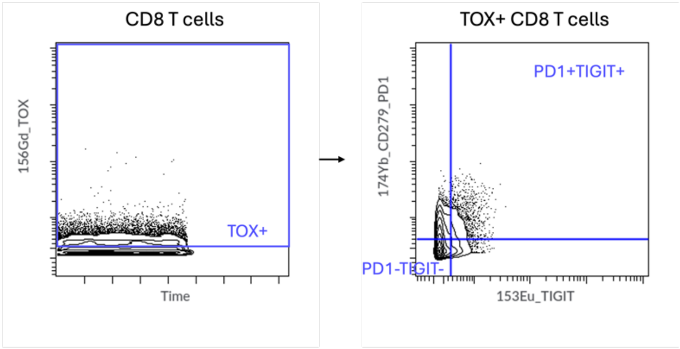
CD8 T cells
156Gd_TOX
TOX+
Time
TOX+ CD8 T cells
174Yb_CD279_PD1
PD1+TIGIT+
PD1-TIGIT-
153Eu_TIGIT

Supplemental Figure 1. Gating strategy for data clean-up and all relevant figures in the manuscript (Figure 3-5), shown for a representative sample. (A) Gaussian gating was performed using the Gaussian paramenters (center, offset, width, and residual). This was followed by bead-removal (140Ce-negative), and gating on cells positive for 191Ir and 193Ir to distinguish single cells from debris and doublets. Dead cells were excluded by gating on cells negative for Cisplatin (195Pt). Finally, gates were set on CD138-negative cells to exclude tumor cells, and positive for CD45 to include all immune cells. (B) To identify monocytes (CD14+) and NK cells (CD56+), cells were gated negative for CD3 and CD20, followed by gating on cells positive for CD14 or CD56. To identify T cells (CD3+) and B cells (CD20+), cells were first gated negative for CD11c, CD56, and CD14. Then T cells and B cells were gated according to expression of CD3 and CD20. T cells were further gated into CD8 and CD4 T cells, and regulatory T (T_{reg}) cells (CD4+CD25+FoxP3+) were gated from CD4 T cells according to expression of CD25 and FoxP3. (C) To identify T_{CM} (CD45RA-CD45RO+CCR7+), T_{EM} (CD45RA-CD45RO+CCR7-), T_{EMRA} (CD45RA+CD45RO-CCR7-), and naïve CD8 T cells (CD45RA+CD45RO-CCR7+) within the CD8 T cell population, cells were first gated according to expression of CD45RA and CCR7, and then each quadrant was further gated into CD45RO+ and CD45RO- populations. Naïve CD8 T cells were then further gated into CD95+ and CD95- populations. (D) TOX+ T cells were identified by gating CD8 T cells positive for TOX, and this population was then further divided into either double-positive (PD-1+TIGIT+) or double-negative (PD-1-TIGIT-) TOX+ CD8 T cell populations.

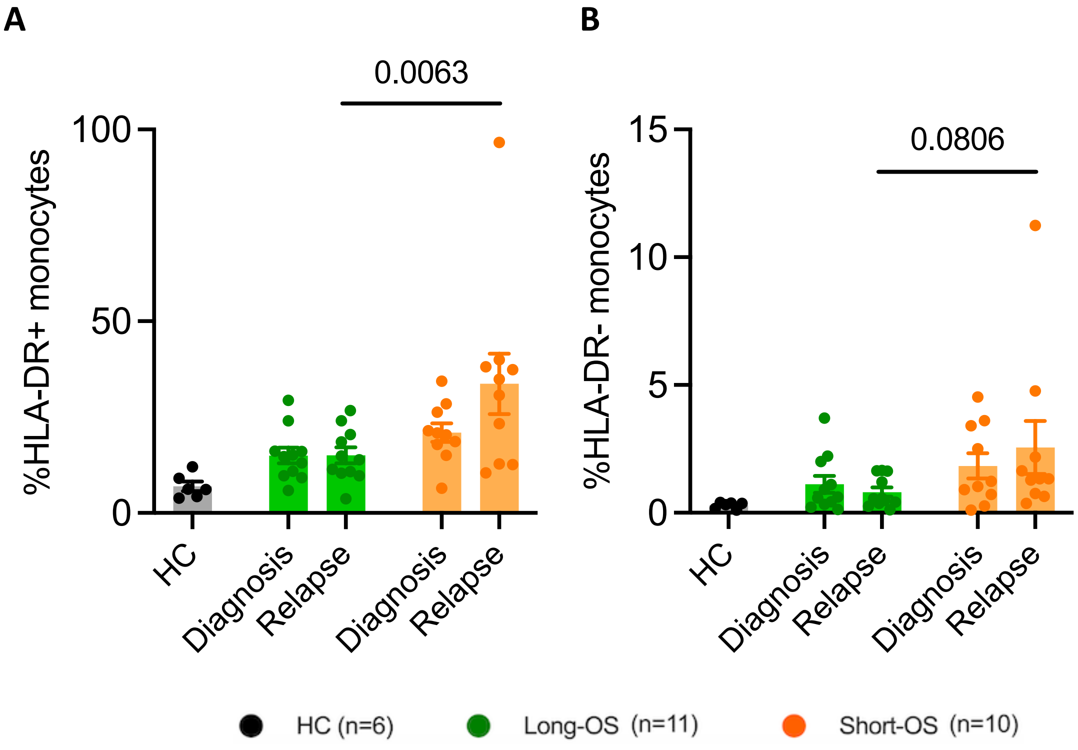

Supplemental Figure 2. Percentage of HLA-DR+ (A) and HLA-DR- (B) monocytes (CD138-CD3-CD20-CD14+) within the live CD45+ population in the BM of myeloma patients and age-matched healthy controls (HC, n=6). Paired diagnosis and last relapse samples from n=11 long-OS patients and n=10 short-OS patients were included. Mean ± SEM shown. Statistical significance was determined by repeated measures two-way ANOVA and Šidák's multiple comparisons test on long-OS and short-OS patient samples. Data from HCs are also shown. Selected P values are indicated.

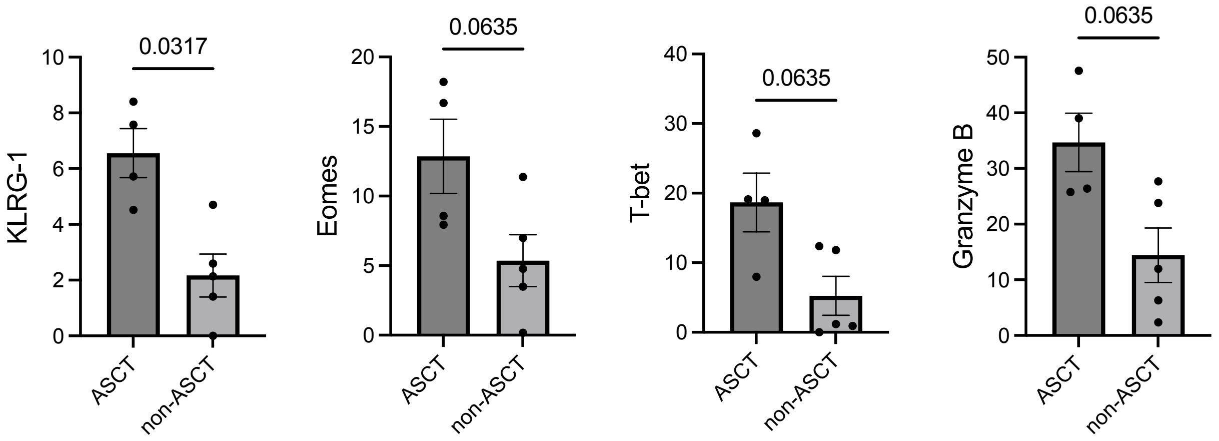

Supplemental Figure 3. Median mass intensity (MMI) of KLRG-1, Eomes, T-bet, and granzyme B within the CD8 T cell population in the BM of short-OS myeloma patients at relapse, subgrouped according to having (ASCT) or not having (non-ASCT) received ASCT. Statistical significance was determined by unpaired, nonparametric Mann-Whitney test. Last relapse sample from short-OS patients shown (ASCT=4, non-ASCT=5). ASCT: autologous stem cell transplantation.

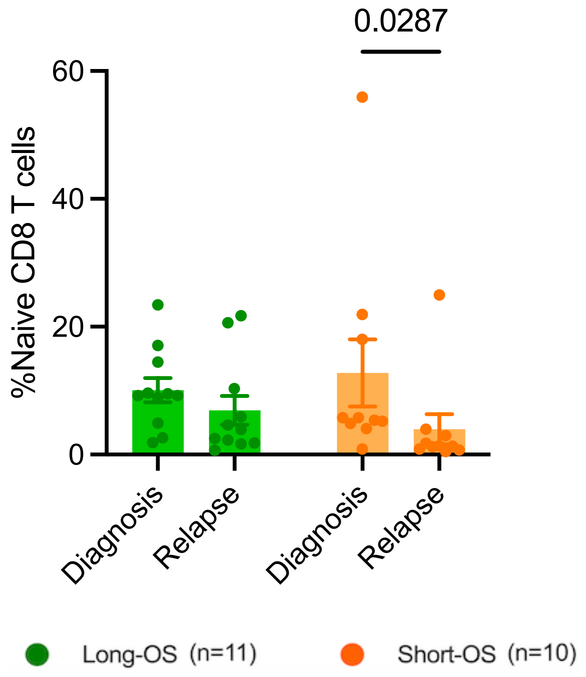

Supplemental Figure 4. Percentage of naïve CD8 T cells, as found by FlowSOM clustering, within the CD8 T cell population. Diagnosis and all available relapse samples were used for FlowSOM clustering. Paired diagnosis and last relapse samples from n=11 long-OS patients and n=10 short-OS patients are included in the bar graph. Mean ± SEM shown. Statistical significance was determined by repeated measures two-way ANOVA and Šidák's multiple comparisons test. P values are indicated where $P < 0.05$.

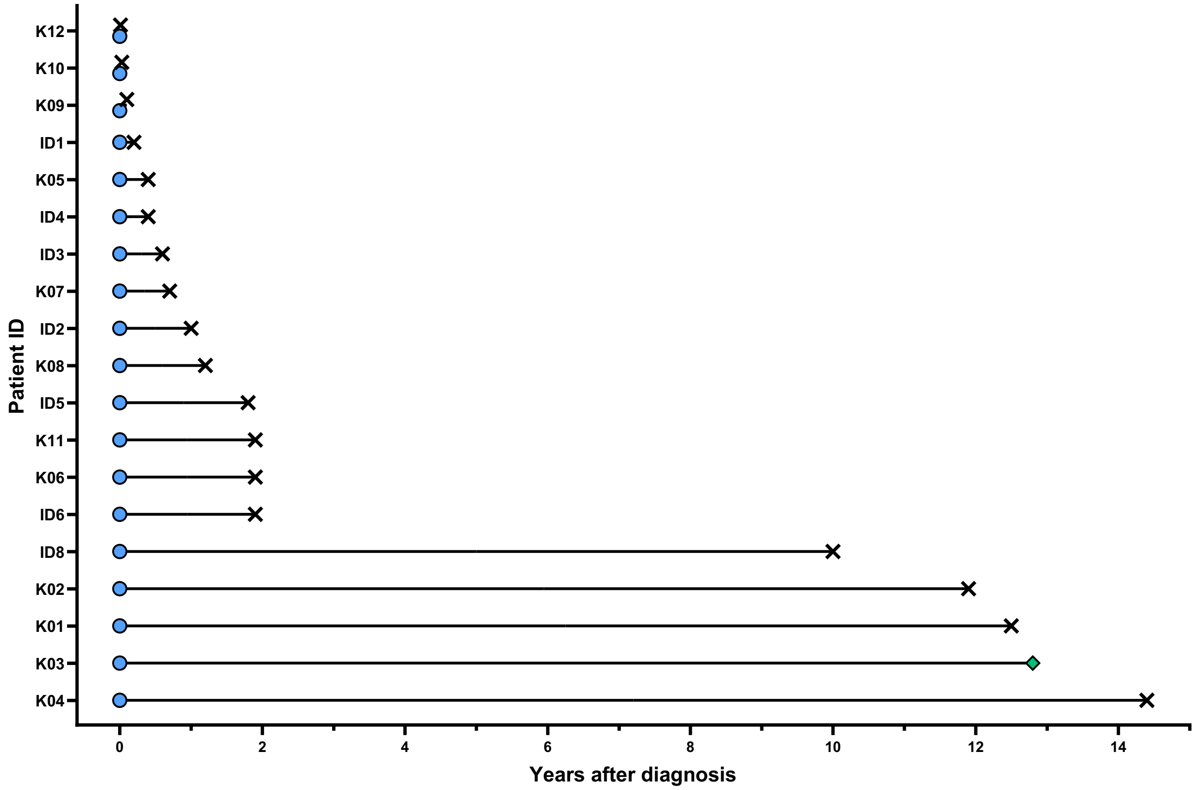

Supplemental Figure 5. Swimmer plot of showing immune assay performed in relation to OS. Includes patients with only samples from diagnosis analyzed. CyTOF was performed on CD138-negative BMMNCs or BMMNCs. Blue: CyTOF; green: alive; cross: dead.

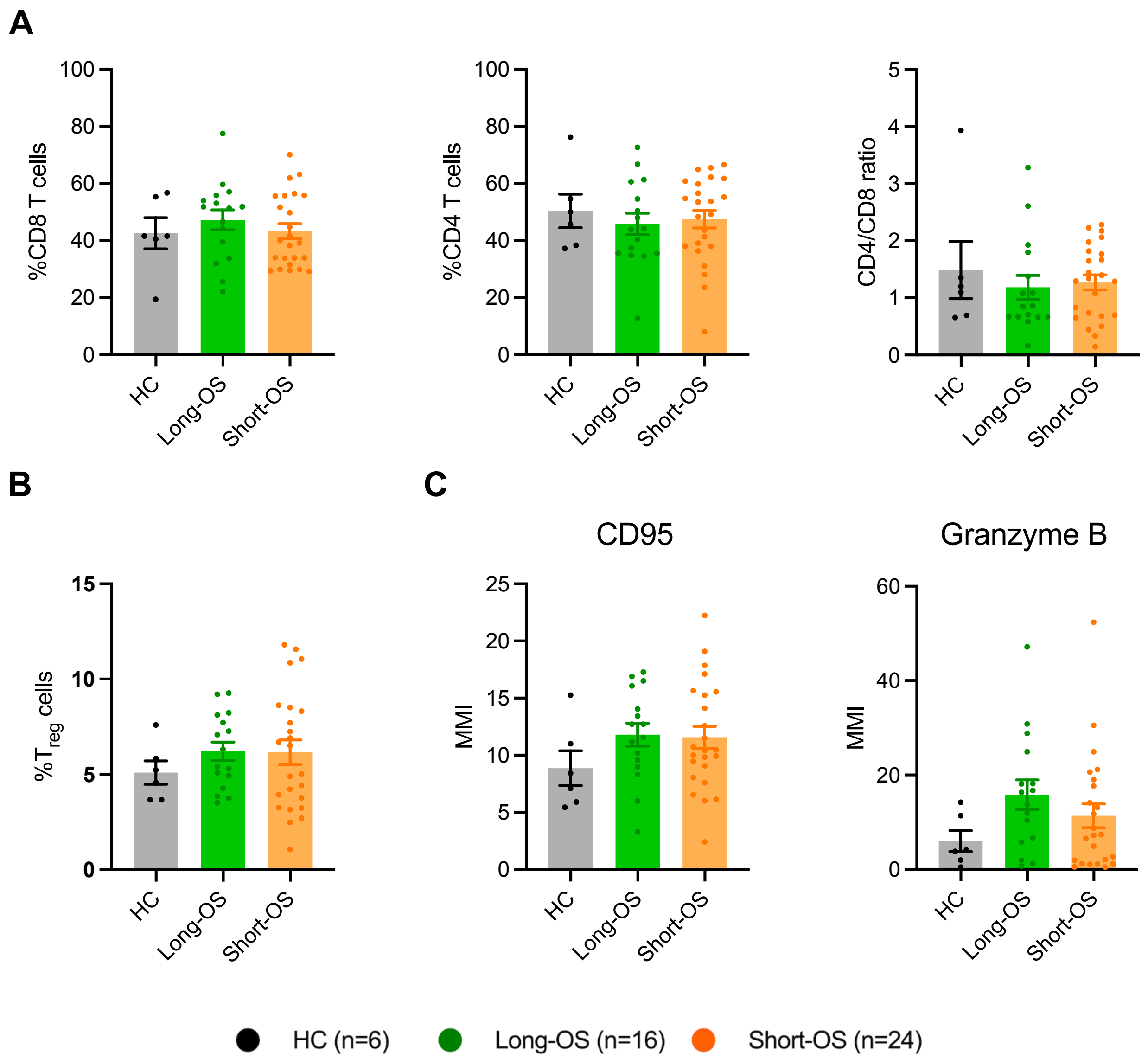

Supplemental Figure 6. Percentage of (A) CD8 and CD4 T cells within the T cell population, as well as CD4/CD8 ratio, (B) regulatory (T_{reg}) cells (CD4+CD25+FoxP3+) within the CD4 T cell population, and (C) median mass intensity (MMI) of CD95 and granzyme B within the CD8 T cell population, in the BM of healthy controls (HC) and myeloma patients at diagnosis. Samples from n=6 HCs, n=16 long-OS patients, and n=24 short-OS patients at diagnosis were included. Mean ± SEM shown.

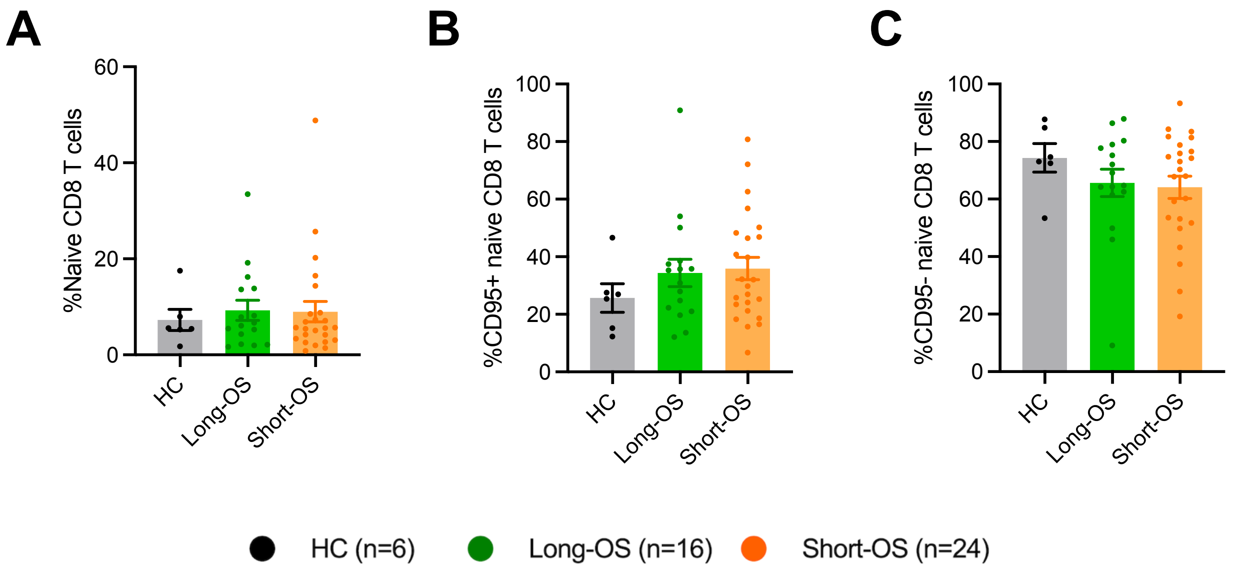

Supplemental Figure 7. Percentage of (A) naïve CD8 T cells (CD45RA+CD45RO-CCR7+), and (B) CD95+ and (C) CD95- naïve CD8 T cells within the naïve CD8 T cell population in the BM of healthy controls (HC) and myeloma patients at diagnosis. Samples from n=6 HCs, n=16 long-OS patients, and n=24 short-OS patients at diagnosis were included. Mean ± SEM shown.
